# Transitions in abortion care in Ghana: revealing the potential of globalizing provider attitudes

**DOI:** 10.1186/1472-6963-14-S2-P5

**Published:** 2014-07-07

**Authors:** Patience Aniteye, Susannah Mayhew, Beverley O’Brien

**Affiliations:** 1 Department of Community Health, School of Nursing, University of Ghana, Legon, Accra, Ghana; 2 Department of Global Health and Development, London School of Hygiene and Tropical Medicine, London, UK; 3 Faculty of Nursing, University of Alberta, Edmonton, Alberta, Canada

## Background

Unsafe abortion remains a public health problem in Ghana with colossal costs to families, communities and health services, evident in persistently high maternal mortality ratios. It is well known that access to and utilization of family planning, safe and legal abortion services and quality post abortion care could help curtail the ramifications of unsafe abortions. Ghana has a liberal abortion law, yet safe, legal abortion services are not easily accessible in public health facilities. The extent to which attitudes of providers are influenced by global discourse on abortion and the role of safe abortion in reducing maternal mortality rates, is not well known.

## Materials and methods

Using a purposive sample of 36 providers, in-depth interviews were carried out to explore the knowledge, interpretation and application by providers of the abortion law and the MOH/GHS policy, and their attitudes and experiences vis-à-vis provision of abortion services.

## Results

Many obstetricians knew the law but pharmacists and midwives were less knowledgeable of the law. The law was perceived as liberal but full of gaps and inconsistencies making its interpretation and application a problem. Obstetricians’ exposure to international treaties and conventions (including WHO statements, ICPD, ICPD + 5, Beijing Conferences) influenced their attitudes towards provision of safe abortion services while midwives’ attitudes were largely affected by their religious inclinations and (local) social context. Lack of resources such as trained personnel who are willing to provide abortion services, unavailability of manual vacuum aspiration kits and the lack of support and cooperation of hospital administrations also influenced provider actions and hampered abortion care. Exposure of health personnel to trends in abortion care in other countries through conferences and treaties did appear to influence how they thought and acted concerning abortion care. Values clarification workshops for health providers (continuing education at which global evidence was considered) were also cited as helping to influence their (positive) attitudes towards women with unplanned pregnancies who need abortion care.

## Conclusions

Breaking the culture of silence surrounding abortion through discussions on the topic, mobilizing health providers who are not conscientious objectors and promoting discourse from international Conferences through continuing education and in public media all hold potential for sustaining comprehensive abortion care in Ghana.

